# Papillary thyroid microcarcinoma with lung metastases: a case report and review of the literature

**DOI:** 10.1186/s13044-021-00106-0

**Published:** 2021-06-11

**Authors:** Tadafumi Shimizu, Takaaki Oba, Tatsunori Chino, Ai Soma, Mayu Ono, Tokiko Ito, Toshiharu Kanai, Kazuma Maeno, Yoshinori Sato, Takeshi Uehara, Ken-ichi Ito

**Affiliations:** 1 Division of Breast and Endocrine Surgery, Department of Surgery, Shinshu University School of Medicine, 3-1-1 Asahi, Matsumoto, Nagano, 390-8621 Japan; 2 Division of Laboratory Medicine, Shinshu University Hospital, Matsumoto, Japan

**Keywords:** Papillary thyroid microcarcinoma, Lung metastasis, Lymph node metastasis, 131-iodine therapy

## Abstract

**Background:**

Distant metastasis from papillary thyroid microcarcinoma (PTMC) is rare. Here we report a case of PTMC with multiple lung metastases.

**Case presentation:**

A 64-year-old man presented to our hospital with abdominal pain. Computed tomography incidentally revealed multiple lung nodules. The lung tumor was histologically diagnosed as metastasis of papillary thyroid carcinoma (PTC) by core needle biopsy via thoracoscopy. The patient was referred to our department for further examination. Neck ultrasonography revealed a 0.9 cm hypoechoic nodule in the right lobe of the thyroid gland, which was diagnosed as PTC by fine-needle aspiration cytology. Subsequently, total thyroidectomy was performed, followed by radioiodine therapy. Iodine-131 (131-I) scintigraphy showed a strong accumulation in the lung metastasis. The patient presented no evidence of progression of lung metastasis for 25 months after the operation.

**Conclusions:**

Lymph node metastasis or extraglandular extension has been reported in the few published cases of metastatic PTMC, including the present case, and the average age of these cases was 58.8 ± 12.0 years. Although active surveillance without surgical resection is expected to become a standard of care for PTMC, this case indicates that a subset of PTMC patients with risk factors may develop distant metastases. Hence, careful preoperative screening is required to avoid complications associated with completion thyroidectomy.

## Background

A papillary thyroid carcinoma (PTC) measuring less than or equal to 1.0 cm in diameter is defined as a papillary thyroid microcarcinoma (PTMC). PTC generally grows slowly, and recent clinical studies have demonstrated that most PTMCs also do not increase in size for long time-periods [1–4]. Given the indolent nature of PTC, distant metastasis from PTMC has been considered very rare [3–5].

Although tumor size, multifocal cancers, extrathyroidal extension, and lymph node metastasis have been suggested as prognostic factors for PTMC recurrence [6–9], the risk factors for distant metastasis remain unclear. Here, we have reported a case of PTMC with multiple lung metastases and have reviewed similar cases in the literature to explore the risk factors for distant metastases from PTMC.

## Case presentation

**Fig. 1:**
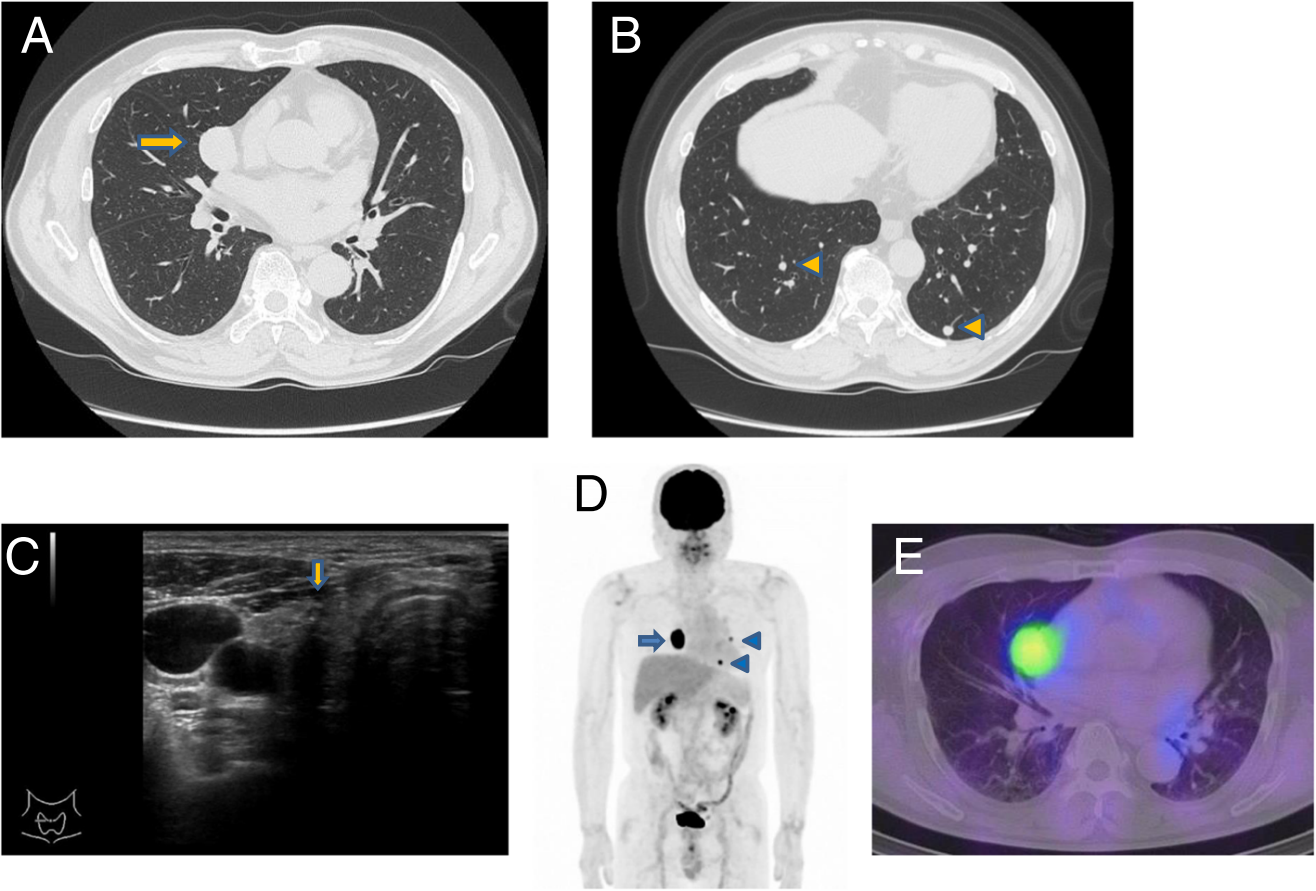
**A**, **B**: Computed tomography findings in the lung. A solid 30 mm mass (arrow) in the middle lobe of the right lung (**A**) and several 5 mm nodules (arrowheads) (**B**) can be seen in both lungs. **C**: Ultrasonographic findings in the thyroid gland. A hypoechoic mass measuring 8.6 × 4.6 mm and showing an indistinct border can be seen in the right lobe of the thyroid gland (arrow). **D**: ^18^F-fluorodeoxyglucose positron emission tomography (FDG-PET) findings. Strong FDG uptake is detected in the right lung tumor (arrow) and multiple tumors in the left lung (arrowhead). **E**: 131-iodine scintigraphy findings after the second radioactive iodine (RAI) treatment. 131-iodine has accumulated in the metastatic tumor in the middle lobe of the right lung

**Fig. 2:**
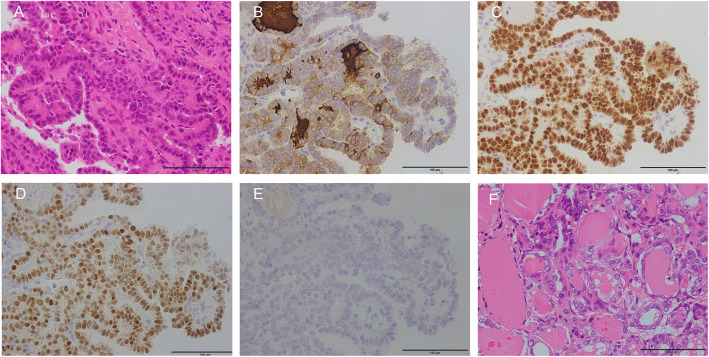
**A**-**E**: Histopathological findings of the tumor in the middle lobe of the right lung. Atypical cells with intranuclear cytoplasmic inclusions have grown with papillary formations (**A**; HE, × 100). The tumor cells are positive for thyroglobulin (**B**; × 100), thyroid transcription factor-1 (**C**; × 100), and PAX8 (**D**; × 100) and negative for Napsin A (**E**; × 100). Scale bar: 100 μm. **F**: Histopathological findings of the thyroid. The tumor in the right lobe of the thyroid is diagnosed as papillary thyroid cancer, consistent with the lung tumor. Firm infiltration into the surrounding fat tissue can be observed (× 100). Scale bar: 100 μm

A 64-year-old man presented to our hospital with abdominal pain. Computed tomography (CT) scan to investigate the cause of abdominal pain incidentally revealed multiple lung nodules. He had no history of malignant tumors. Thoracoabdominal CT revealed a solid tumor in the middle lobe of the right lung measuring 30 mm in diameter (Fig. 1A) and multiple bilateral lung nodules less than 5 mm in size (Fig. 1B). Primary lung cancer or multiple lung metastases from a malignant tumor originating in another organ was suspected. Because the lesions were difficult to access via a transbronchial approach, core needle biopsy of the tumor in the middle lobe of the right lung was performed via thoracoscopy. Histopathological examination revealed atypical cells with intranuclear cytoplasmic inclusions that formed papillary structures (Fig. 2A). In addition, immunohistochemical examination showed positive staining for thyroglobulin (Tg), thyroid transcription factor-1 (TTF-1), and PAX8, but negative staining for Napsin A (Fig. 2B–E). Based on these findings, the tumor was diagnosed as a metastasis of PTC.

Subsequently, the patient was closely examined for a primary lesion in the thyroid gland. Physical examination showed no palpable nodule on the neck. Although Tg antibody was positive, the serum Tg level was not elevated. Thyroid peroxidase antibody was negative [2.1 IU/mL (≤ 5.2)]. (Table 1). Ultrasonography revealed a hypoechoic nodule with an indistinct border measuring 0.9 × 0.5 cm in the right lobe of the thyroid gland (Fig. 1C). Multiple lung nodules showed strong uptake on ^18^F-fluorodeoxyglucose positron emission tomography (FDG-PET), but no uptake was detected in the thyroid gland tumor (Fig. 1D). As fine needle aspiration cytology from the thyroid nodule demonstrated atypical cells with intranuclear cytoplasmic inclusions and grooved nuclei, the nodule was diagnosed as PTC. Consequently, the patient was diagnosed with PTMC with multiple lung metastases and underwent total thyroidectomy with central neck lymph node dissection.

**Table 1:** Pre-operative blood test findings of the 64-year-old man with papillary thyroid microcarcinoma and lung metastases

Parameter	Normal range	Before surgery
Free T3 (pg/ml)	2.30–4.00	3.10
Free T4 (pg/ml)	0.9–1.7	1.36
TSH (μIU/ml)	0.50–5.00	2.27
Tg (ng/ml)	≤ 33.7	31.0
Tg Ab (IU/ml)	≤ 40.6	53.9
TPO Ab (IU/ml)	≤5.2	2.1

*TSH* thyroid stimulating hormone, *Tg Ab* thyroglobulin antibody, *TPO Ab* thyroid peroxidase antibody, *Tg* thyroglobulin

Histopathological examination of the resected specimen revealed that the thyroid tumor in the right lobe was PTC 0.7 × 0.3 cm in size (pT1a) with metastases to the paratracheal lymph nodes (pN1a). Histopathological findings of the tumor in the right lobe and metastatic lymph nodes were consistent with those observed in the lung tumor (Fig. 2F). Extrathyroid extension to the perithyroid soft tissue was detected (pEx1). Extranodal extension of the metastatic lymph nodes was not detected. The histopathological stage was Stage IVB [10]. Three months after the operation, the patient received radioactive iodine (RAI) therapy after withdrawal of thyroid hormone therapy for three weeks. A total of 3700 MBq (100 mCi) of RAI was administered for the initial therapy, and then 131-I scintigraphy was performed eight days later. In 131-I scintigraphy following the first RAI therapy, an accumulation of 131-I was observed in the thyroid bed alone. However, in the 131-I scintigraphy following the second and third therapies a strong 131-I accumulation was observed in the metastatic lesion in the right lung with the same amount of RAI (Fig. 1E). The patient’s serum Tg level under TSH suppression did not change considerably when compared with that before the RAI treatment (Table 2). He has been well with no signs of progression in the lung and other organs 25 months after the operation.

**Table 2:** Postoperative blood test findings of the 64-year-old man with papillary thyroid microcarcinoma and lung metastases

Parameter	Normal range	3 M after 1st RAI treatment	3 M after 2nd RAI treatment	3 M after 3rd RAI treatment
TSH (μIU/ml)	0.50–5.00	0.005	0.007	0.005
Tg (ng/ml)	≤ 33.7	17.3	22.6	24.6
Tg Ab (IU/ml)	≤ 40.6	21.5	12.9	14.4

*M* months, *TSH* thyroid stimulating hormone, *Tg Ab* thyroglobulin antibody, *Tg* thyroglobulin,

*RAI* radioactive iodine

## Discussion and conclusions

The overall incidence of PTC continues to increase worldwide [11–14]. In addition, PTMC has increased due to the spread of health screening, technological progress in ultrasonography, and improved accuracy of ultrasonography-guided fine needle aspiration cytology [15–17]. Although PTCs generally grow slowly, lymph node metastases are known to occur even when the primary tumor is small. As for PTMC, Elliot et al. reported that 11.5% of PTMC had cervical lymph node metastases in a retrospective study of 112 resected cases [18]. However, two prospective observational studies from Japan have reported that the incidence of distant metastasis from PTMC was very rare [3, 4]. Ito et al. reported that no patients developed distant metastasis for ten years after the diagnosis of PTMC in a study of 1235 patients observed without surgery [3]. Similarly, Sugitani et al. reported that no distant metastasis occurred in 230 non-surgical PTMC patients who were observed for ten years [4].

However, in a large-scale retrospective analysis using the Surveillance, Epidemiology and End Results (SEER) Cancer Database (18,445 cases) in the United States, Yu et al. reported that the incidence of distant metastasis from PTMC was 0.5% [19]. In a recent retrospective analysis of 8808 patients with PTMC in Korea, Jeon et al. reported that 12 patients (0.1%) had distant metastases, and four patients died of the primary disease [20]. Thus, although distant metastases from PTMC rarely occur, PTMC should be included in the differential diagnosis list for the primary lesion when metastatic lesions from unknown origins are encountered.

To identify risk factors for distant metastases from PTMC, we searched the literature for cases of distant metastasis from PTMC, which were confirmed by histological examination or RAI uptake over the past 20 years and found 24 such cases (Table 3) [5, 15, 20–29]. In the analysis of the SEER cancer database reported by Yu et al. [19], there were 91 cases of distant metastases from PTMC; however, detailed clinical information of each patient was not reported. Therefore, we excluded these cases from our present analysis. It has been generally considered that tumor size, extraglandular extension of the primary tumor, extranodal extension of metastatic lymph nodes, size of metastatic lymph nodes, older age, and male sex are possible risk factors for recurrence of PTC [30–33]. In addition, the recently revised TNM staging system defined a high risk for people who were ≥ 55 years of age [10]. Jeon et al. recently reported the association of disease-specific mortality with old age, large metastatic lymph nodes with extranodal extension, and a change to an aggressive pathologic subtype of metastatic lymph nodes by analyzing a large number of patients with PTMC [20]. Although detailed information on pathological diagnosis was not stated for several of the 24 cases found in the literature, either lymph node metastasis or extraglandular extension was observed in most patients, including the present case, and the average age of these cases was 58.8 ± 12.0 years. Thus, the possibility of distant metastases should be considered in PTMC cases with risk factors for PTC recurrence.

**Table 3:** Clinicopathological features of previously reported cases of papillary thyroid microcarcinoma with distant metastasis

No	Author Year	Age (years) Sex	Reasons for detection	Diagnostic method for PTMC metastases	Surgery for thyroid	Tumor size (cm)	pN	Ex	Metastatic site	131-I therapy	Prognosis
1	Murakami, et al. , 2001 [5]	63 M	Abdominal tumor	Extirpation of abdominal tumor	Partial resection of L thyroid lobe and ND	0.1	+	N. A	Rectus muscle	–	1 y 8 m, alive
2	Yamada, et al., 2001 [14]	68 M	Chest X ray	Fiberoptic bronchoscopic biopsy	TT	< 1.0	N. A	N. A	Lung	+	1 y 10 m, alive
3	Erdem, et al., 2003 [20]	40 M	Dysphagia	Resection of a R parapharyngeal mass	TT and ND	0.8	–	N. A	Parapharyngeal	+	3 y, alive
4	Liou, et al., 2005 [21]	50 F	Right pelvic fracture	R hemipelvectomy and renal biopsy	TT	1	N. A	N. A	Kidney, pelvic bone, lung	+	about 3 y, alive
5	Itoh, et al. 2008 [22]	82 F	Cough, back pain	Fiberoptic bronchoscopic biopsy	None	0.6	N. A	+	Brain, liver, pancreas, kidney, ovary, bone, lung	–	7 m, dead
6	Lecumberri, et al., 2010 [23]	65 F	Headache, tinnitus	Resection of a cerebellar mass	TT and ND	0.2	–	+	Brain	+	7 y, dead
7	Xu, et al. 2011 [24]	46 F	Cervical mass	131-I WBS and SPECT/CT	TT and ND	0.3	+	N. A	Brain, lung	+	3 m, alive
8	Saito, et al., 2011 [25]	70 F	Cough	R lung lobectomy	Subtotal thyroidectomy and ND	0.8	–	N. A	Lung	–	3 y 10 m, alive
9	Kozu, et al. 2014 [26]	70 M	Chest CT	L lung lobectomy	None	N. A	N. A	N. A	Lung	–	4 m, alive
10	Kaseda, et al., 2016 [27]	66 F	Chest CT	Transbronchial biopsy and R lung lobectomy	None	N. A	N. A	N. A	Lung	–	N. A
11	Kawai, et al., 2016 [28]	In 70’s M	Chest discomfort	R lung lobectomy	R thyroid lobectomy and ND	1	+	+	Lung	–	11 y, alive
12	Jeon, et al., 2016 [19]	51 F	Neck US	131-I WBS and SPECT/CT	TT and ND	0.8	+	–	Lung	+	1.9 y, alive
13	Jeon, et al., 2016 [19]	31 F	Neck US	131-I WBS and SPECT/CT	TT and ND	0.9	+	+	Lung, bone	+	6.7 y, alive
14	Jeon, et al., 2016 [19]	55 F	Neck US	131-I WBS and SPECT/CT	TT and ND	0.9	+	+	Lung, bone	+	7.6 y, alive
15	Jeon, et al., 2016 [19]	59 F	Neck US	131-I WBS and SPECT/CT	TT and ND	1	+	+	Lung	+	2.1 y, alive
16	Jeon, et al., 2016 [19]	73 F	Neck US	131-I WBS and SPECT/CT	TT and ND	1	+	+	Lung	+	8.8 y, alive
17	Jeon, et al., 2016 [19]	54 F	Hoarseness	131-I WBS and SPECT/CT	TT and ND	0.8	+	+	Lung	+	10.7 y, alive
18	Jeon, et al., 2016 [19]	63 F	Neck mass	131-I WBS and SPECT/CT	TT and ND	0.8	+	+	Lung	+	11.4 y, dead
19	Jeon, et al., 2016 [19]	46 F	Chest CT	131-I WBS and SPECT/CT	TT and ND	0.7	+	+	Lung, bone	+	15 y, alive
20	Jeon, et al., 2016 [19]	65 M	Hoarseness	131-I WBS and SPECT/CT	TT and ND	0.6	+	–	Lung, bone	+	1 y, dead
21	Jeon, et al., 2016 [19]	58 M	Neck mass	131-I WBS and SPECT/CT	TT and ND	1	+	–	Lung, brain	+	4.9 y, dead
22	Jeon, et al., 2016 [19]	60 F	Neck mass	131-I WBS and SPECT/CT	TT and ND	0.8	+	–	Lung, brain	+	10 y, dead
23	Jeon, et al., 2016 [19]	63 F	Neck mass	131-I WBS and SPECT/CT	TT and ND	0.9	+	+	Lung, bone	+	10.7 y, dead
24	Our case	64 M	Chest CT	Lung biopsy via VATS	TT and ND	0.7	+	+	Lung	+	2 y 1 m, alive

*pN* pathological cervical lymph node metastases, *Ex* extrathyroid extension, 1*31-I* 131-iodine, *WBS* whole body scan,

*SPECT/CT* single-photon emission computed tomography, *CT* computed tomography, *VATS* video-assisted thoracic surgery, *TT* total thyroidectomy, *ND* neck dissection, *N.A* not available, *y* years, *m* months

Recently, Song et al. identified Mesenteric Estrogen Dependent Adipogenesis (MEDAG) as one of the genes associated with lymph node metastases and poor disease-free survival of patients with PTMC by analyzing the microarray data from The Cancer Genome Atlas [34]. Although the significance of MEDAG in the onset of distant metastasis has not been elucidated, it is expected that predictive factors of distant metastasis will be identified by multigene analyses of a large number of cases in the near future.

RAI therapy is the standard therapy for distant metastases from PTC, and patients must undergo total thyroid gland resection before treatment. However, it is known that completion thyroidectomy is associated with a higher incidence of surgical complications, such as recurrent laryngeal nerve paralysis and permanent hypoparathyroidism, compared with initial surgery [35]. To avoid such complications due to re-operation, PTC patients should be carefully assessed for distant metastases by CT or other imaging modalities before surgery, and if there are distant metastases, total thyroidectomy should be performed in the first surgery.

However, Kawano et al. recently showed that routine chest CT at the time of PTMC diagnosis did not identify distant lung metastasis in 1000 patients with low-risk PTMC and suggested that chest CT is not beneficial for patients with PTMC in relation to factors such as cost, radiation exposure [36]. Considering these findings, when cervical ultrasonography reveals the existence of extrathyroidal extension of the primary lesion, extranodal extension of the metastatic lymph nodes, or large metastatic lymph nodes in patients over 55 years of age, we should consider screening for distant metastases to avoid complications from reoperation and to facilitate subsequent RAI therapy.

In conclusion, PTMC with multiple lung metastases is rare. Although active surveillance of PTMC is expected to remain the standard for care, this case suggests that a subset of PTMC patients may develop distant metastases. In particular, screening for distant metastases should be considered in patients with risk factors for recurrence of PTC.

## Data Availability

The datasets used during the current study are available from the corresponding author on reasonable request.

## Abbreviations

PTC: papillary thyroid carcinoma
PTMC: papillary thyroid microcarcinoma
131-I: Iodine-131
CT: Computed tomography
Tg: Thyroglobulin
TTF-1: Thyroid transcription factor-1
FDG-PET: ^18^F-fluorodeoxyglucose positron emission tomography
Tg Ab: Thyroglobulin antibody
TSH: Thyroid stimulating hormone
TPO Ab: Thyroid peroxidase antibody
RAI: Radioactive iodine
SEER: Surveillance, Epidemiology and End Results
MEDAG: Mesenteric Estrogen Dependent Adipogenesis
